# Physics at the Cutting Edge: The Essential Science Behind Thoracic Surgery

**DOI:** 10.3390/jcm13226752

**Published:** 2024-11-09

**Authors:** Luca Bertolaccini, Virginia Piva, Antonio Mazzella, Monica Casiraghi, Marco Maria Jacopo Felisi, Lorenzo Spaggiari

**Affiliations:** 1 Department of Thoracic Surgery, IEO, European Institute of Oncology IRCCS, 20141 Milan, Italylorenzo.spaggiari@ieo.it (L.S.); 2 Department of Physics, University of Milan, 20122 Milan, Italy; virginia.piva@unimi.it; 3 Department of Medical Physics, ASST GOM Niguarda, 20162 Milan, Italy; marcomariajacopo.felisi@ospedaleniguarda.it; 4 Department of Oncology and Hemato-Oncology, University of Milan, 20122 Milan, Italy

**Keywords:** thoracic surgery, lung cancer, radiosterilization, electrosurgery, fluid dynamics, medical physics, surgical technology

## Abstract

Thoracic surgery is deeply intertwined with the principles of physics, which govern the tools and techniques used in various procedures. A thorough understanding of these principles is essential for the safe and effective use of surgical technology, advancing surgical techniques, and developing new medical devices. This manuscript provides a comprehensive overview of crucial physical principles relevant to thoracic surgery, such as radiosterilization, electrosurgery, fluid dynamics, endoscopic techniques, diffusion principles, and laser technologies. This manuscript aims to enhance thoracic surgeons’ understanding of how physics underpins their practice by elucidating the connections between these principles and their medical applications. This multidisciplinary approach seeks to improve surgical outcomes by fostering a deeper appreciation of the fundamental science behind thoracic surgery, thereby encouraging innovation and the safe, effective use of advanced surgical technologies.

## 1. Introduction

The practice of thoracic surgery, like all branches of modern medicine, is deeply intertwined with the principles of physics. While the immediate goal of surgery is to alleviate disease and restore health, the tools and techniques employed in thoracic procedures are fundamentally governed by the laws of physics. A thorough understanding of these principles is essential for the safe and effective use of surgical technology, the advancement of surgical techniques, and the development of new medical devices.

Thoracic surgeons must frequently engage with a variety of physical phenomena. From the radiosterilization of surgical instruments, which ensures a sterile operating environment, to electrosurgical devices that rely on electrical currents to cut tissue or coagulate blood, each aspect of their practice is grounded in the physical sciences. In addition, the principles of diffusion and fluid dynamics and the behavior of light in endoscopic procedures are essential to thoracic surgery. Fluid dynamics, governed by laws such as Poiseuille’s law and Bernoulli’s principle, underpins the understanding of blood flow, which is crucial for managing conditions such as aneurysms or ensuring proper circulation during surgery. Using lasers and endoscopic tools allows for minimally invasive procedures, reducing the patient recovery time and improving outcomes.

This manuscript aims to provide a comprehensive overview of the physical principles forming thoracic surgery’s foundation. By exploring the applications of these principles in the context of radiosterilization, electrosurgery, fluid dynamics, and other related fields, we hope to enhance the understanding and practice of thoracic surgeons. A deeper appreciation of these underlying mechanisms will improve the safety and efficacy of thoracic surgical procedures and inspire innovation in surgical techniques and technology.

## 2. Radiosterilization

Sterilization in thoracic surgery ensures patient safety and prevents postoperative infections. This process encompasses various techniques and protocols for maintaining a sterile environment during surgical procedures involving the thoracic cavity. Due to the complexity of the procedures and the sensitive nature of the organs involved, the risk of infection in thoracic surgery is significant. Infections can lead to severe complications, including pneumonia, sepsis, and prolonged hospital stays.

Therefore, stringent sterilization protocols are essential to minimize these risks. Effective sterilization practices in thoracic surgery are fundamental to patient safety and successful surgical outcomes. Sterilization protocols in thoracic surgery can vary significantly based on the specific procedure type [1,2,3].

### The Physics Behind 

Radiosterilization leverages radiation-induced damage to sterilize surgical equipment, implants, and other medical devices before procedures [4,5]. The interaction of ionizing radiation with cellular material primarily involves indirect effects. When sufficient energy is delivered in a single event, secondary high-energy electrons known as delta rays are produced. Upon crossing DNA, these rays can induce various types of damage, such as single-strand breaks (SSBs) and double-strand breaks (DSBs). While SSBs involve the breakage of one DNA strand and can often be repaired by the cell, DSBs involve both strands being severed and are significantly more lethal as they are difficult for the cell to repair. The accumulation of irreparable DNA damage leads to cell inactivation or death, the basis for the sterilization effect [6]. When cells are exposed to radioactive sources, the ionizing radiation can interact with the cell DNA, causing damage that the cell cannot heal. Ionizing radiation can penetrate the organism, causing the excitation or ionization of its atoms. When enough energy is delivered in a single strike, delta rays are put into motion. Delta rays are high-energy electrons that can produce damage inside the cell. The effect of ionizing radiation on the cell is thus indirect, leaving delta rays to do the hard work. The more the DNA is damaged, the less the cell can summon help to repair the damage, eventually leading to cell inactivation or cell killing. In radiobiology, the survival of cells or organisms exposed to radiation is typically modeled using an exponential or a linear-quadratic model. The exponential model assumes that each organism has a certain probability of being killed by radiation, and this probability is constant per unit dose; the survival fraction *S*(*D*) of this model is therefore described as follows:

(1) $$S\left(D\right)=e^{-\alpha D},$$

where *D* is the dose received by the organisms, and *α* is a constant that represents the population’s sensitivity.

The linear-quadratic model, which is often used to describe cell survival in radiation therapy contexts, extends this concept to include a quadratic term ruled by the constant β:

(2) $$S\left(D\right)=e^{-\alpha D-\beta D^{2}}.$$

Both models imply that complete sterilization (achieving 100% cell death) is theoretically impossible due to the statistical nature of survival curves. However, radiosterilization aims to achieve a sterility assurance level (SAL) of 10^−6^, corresponding to a probability of no more than one viable microorganism per one million sterilized items. A significant advantage of radiosterilization is that the products do not become radioactive post-irradiation up to doses of 10 kGy, ensuring their safety for clinical use.

## 3. Electrosurgery and Electrocauterization

Electrosurgery and electrocoagulation are critical techniques in thoracic surgery, primarily used for cutting tissue and controlling bleeding. These methods utilize electrical currents to achieve surgical goals, enhancing precision and safety during procedures. Electrosurgery involves the use of high-frequency electrical currents to cut or coagulate tissue. Bipolar electrosurgery uses two electrodes to pass the current through the tissue, minimizing thermal spread and allowing for precise control. It is advantageous in delicate areas, such as the thoracic cavity, where the surrounding structures must be preserved. In monopolar electrosurgery, a single active electrode is used, and the current returns through a grounding pad placed on the patient’s body. Monopolar devices effectively cut and coagulate larger areas but carry a higher risk of thermal injury to surrounding tissues. Electrosurgery allows thoracic surgeons to make precise cuts and coagulate tissues with minimal collateral damage. This precision is essential in thoracic surgery, where delicate structures, such as blood vessels, nerves, and the lungs, must be carefully navigated.

Electrocoagulation is a specific application of electrosurgery focused on hemostasis. It involves the application of an electrical current to coagulate blood vessels, effectively sealing them and preventing excessive bleeding.

Direct coagulation applies the current directly to the vessel, causing immediate coagulation. It is beneficial in controlling bleeding during thoracic surgeries. Inductive coagulation uses a higher frequency to induce coagulation without significant thermal damage to the surrounding tissues. It is advantageous in sensitive areas.

These techniques effectively coagulate blood vessels, significantly reducing intraoperative bleeding. Minimizing blood loss enhances the surgeon’s visibility, allowing for more the precise dissection and manipulation of tissues.

Modern electrosurgical techniques, especially bipolar electrosurgery, minimize thermal spread to surrounding tissues. This characteristic is vital in thoracic surgery, where excessive heat can damage adjacent organs and structures, leading to complications. Electrosurgical devices can be used in various thoracic procedures, including lung resections and mediastinal surgeries. Their versatility allows for multiple applications within a surgical setting, improving the overall efficiency and reducing the operation time. By minimizing trauma and bleeding, electrosurgery can lead to quicker patient recovery times [7,8,9].

Ultrasound technology, particularly ultrasonic scalpels, has become increasingly valuable in surgical procedures, including thoracic surgery. Using ultrasonic devices allows for precise cutting and coagulation, reducing the risk of damage to adjacent tissues. This precision is particularly beneficial in thoracic surgery, where structures such as blood vessels and nerves are closely packed. Compared to traditional electrosurgical devices, ultrasonic scalpels produce less thermal damage to surrounding tissues. This is due to the focused energy delivery and the ability to control the temperature more effectively, which can lead to improved healing and reduced postoperative complications. Ultrasonic scalpels effectively achieve hemostasis during surgical procedures. The coagulation effect is immediate and essential in thoracic surgery, where bleeding control is critical. Using ultrasonic devices supports minimally invasive surgical techniques, such as video-assisted thoracoscopic surgery (VATS). These techniques can lead to shorter recovery times, less postoperative pain, and reduced hospital stays. Studies have shown that using ultrasonic scalpels can lead to lower rates of complications, such as seromas and hematomas, compared to traditional methods. Additionally, patients often experience less postoperative pain, which can enhance recovery [10].

### The Physics Behind 

Electrosurgery knives operate based on heat production, a process governed by Ohm’s law and Joule’s effect, as the electric current flows through the patient’s tissues. Ohm’s law describes the relationship between the voltage (*V*), current (*I*), and resistance (*R*) in an electrical circuit:

(3) V=IR.

This law implies that the current flowing through a conductor between two points is directly proportional to the voltage across the two points and inversely proportional to the conductor’s resistance.

Joule’s first law further explains this process by quantifying the amount of heat produced in a conductor due to the passage of an electric current, expressed as follows:

(4) $$Q=I^{2}Rt,$$

where *Q* is the heat energy produced, *I* is the electric current, *R* is the tissue’s resistance, and *t* is the time the current flows. In electrosurgery, the current flowing through the knife’s circuit is pulsed, which prevents neuromuscular stimulation. The *faradic effect* refers to the physiological responses produced by low-frequency electrical currents, typically between 50 and 100 Hz, which can cause muscle contractions and cardiac stimulation. However, electrosurgical knives operate at high frequencies, usually greater than 300 kHz. This avoids these adverse effects by minimizing the faradic response due to the reduced stimulation of neuromuscular tissues at these higher frequencies.

Electrosurgical ablation, a standard method in thoracic surgery [11], often employs radiofrequency (RF) energy, which generates sufficient heat at targeted sites to achieve the desired tissue ablation.

The term “radiofrequency” refers to an electromagnetic wave with a frequency range from 300 kHz to 3 GHz. This range overlaps with the frequencies used in AM radio transmission but operates at significantly higher power densities in medical applications.

As the term suggests, electromagnetic waves consist of oscillating electric and magnetic fields, which oscillate perpendicularly to each other and the direction of wave propagation. When an RF signal is applied near electrically charged or polarized molecules or ions within tissues, these molecules attempt to align with the rapidly changing direction of the RF field, causing them to vibrate. This vibration generates heat through friction, which, in turn, causes tissue heating. In biological tissues, this induced heating can result in rapid cell death, as higher temperatures cause irreversible cellular damage. This principle of converting RF energy into localized heat is central to the function of electrosurgical devices, allowing precise cutting, coagulation, and tissue destruction while minimizing thermal spread and collateral damage to surrounding tissues.

In addition to standard electrosurgical methods, ultrasonic scalpels have emerged as a valuable tool in thoracic surgery, offering a precise, low-thermal approach to tissue dissection and coagulation in the last few years. The ultrasonic scalpel operates based on mechanical rather than thermal action, allowing for precise tissue dissection while minimizing blood loss. Unlike traditional electrosurgical tools that rely on high temperatures to cut and coagulate tissue, the ultrasonic scalpel uses high-frequency vibrations produced by a piezoelectric crystal. Piezoelectricity is a property observed in certain materials, such as quartz and lead zirconate titanate, where an applied electric field induces mechanical deformation. When an alternating current is applied to the piezoelectric crystal in the scalpel, it rapidly oscillates, producing ultrasonic vibrations around 55–60 kHz frequencies. These vibrations are transmitted to the tip of the scalpel, which, upon contacting tissue, induces molecular friction that severs cells without excessive heat generation. This process enables mechanical coagulation, where the vibrations cause protein denaturation and seal small blood vessels through controlled pressure and friction rather than intense thermal energy. By relying on these precise mechanical forces, the ultrasonic scalpel minimizes the risk of thermal damage to surrounding tissues, making it ideal for procedures where accuracy and tissue preservation are critical.

## 4. Diffusion

The concept of the diffusion law in thoracic surgery primarily relates to the principles governing the movement of gases and fluids within the thoracic cavity, particularly concerning the pulmonary function and managing various thoracic conditions.

In thoracic surgery, diffusion is critical for understanding how oxygen and carbon dioxide are exchanged in the lungs. The efficiency of this process is influenced by factors such as the alveoli’s surface area, the alveolar–capillary membrane’s thickness, and the gases’ partial pressures. Surgical interventions, such as lung resections or transplants, can significantly alter the normal diffusion processes. For instance, after a lobectomy, the remaining lung tissue must compensate for the loss of surface area, which can affect the overall gas exchange efficiency [12]. In pulmonary function testing, diffusion refers to the process by which gases move across the alveolar–capillary membrane in the lungs. Fick’s law states that the rate of the diffusion of a gas is proportional to the surface area available for diffusion and the difference in the partial pressures of the gas across the membrane, and it is inversely proportional to the thickness of the membrane. The diffusion capacity of the lungs for carbon monoxide (DLCO) is a test that measures how gases can diffuse from the alveoli into the bloodstream. It assesses the integrity of the alveolar–capillary membrane and is influenced by the alveolar surface area (an increased surface area enhances the diffusion capacity), membrane thickness (conditions that thicken the membrane, e.g., pulmonary fibrosis, a reduced diffusion capacity), and hemoglobin levels (carbon monoxide binds to hemoglobin so that lower hemoglobin levels can decrease the DLCO) [13]. The balance between ventilation (air reaching the alveoli) and perfusion (blood flow to the alveoli) is crucial for optimal gas exchange. An imbalance can lead to areas of the lung that are well ventilated but poorly perfused (high ventilation/perfusion ratio) or well perfused but poorly ventilated (low ventilation/perfusion ratio), affecting the overall diffusion efficiency [14].

### The Physics Behind 

Diffusion in physical systems is ruled by Fick’s laws of diffusion. Fick’s first law describes diffusion in steady-state systems, where the concentrations do not change over time, while Fick’s second law applies to systems where the concentration varies with time.

Fick’s first law states that the diffusion flux is proportional to the negative gradient of the concentration, and it is mathematically expressed as follows:

(5) $$J=-D(\frac{dC}{dx}),$$

where *J* is the diffusion flux, which represents the amount of substance that flows through a unit area per unit time (e.g., mol/m^2^s). *D* is the diffusion coefficient or diffusivity, which measures how easily particles diffuse through the medium (e.g., m^2^/s). *C* is the concentration of the diffusing substance (e.g., mol/m^3^), and x is the position. The negative sign indicates that diffusion occurs in the direction of decreasing concentration, with the flux magnitude directly proportional to the concentration gradient.

Fick’s second law of diffusion describes time-dependent changes in the concentration and is given as follows:

(6) $$\frac{\partial C}{\partial t}=D\frac{\partial^{2}C}{\partial x^{2}}.$$

In this equation, $\;\frac{\partial C}{\partial t}$ represents the rate of the change in the concentration with time, while $\frac{\partial^{2}C}{\partial x^{2}}$ is the second spatial derivative of the concentration, describing how the concentration gradient itself changes with the position. This law explains how the concentration evolves over time as the system moves towards equilibrium, where concentration differences are minimized, and the flux ideally approaches zero. We can intuitively explain this by considering a system in which a specific molecule diffuses from an area of high concentration to an area of low concentration. As it moves, the concentration itself changes with time, with more molecules that reach the area of low concentration, resulting in a gradual reduction in the concentration difference between the two areas. As the concentration changes over time, so does the concentration gradient (the first derivative of the concentration with respect to the position). This gradient is not uniform; it varies from one point to another within the system, leading to a changing diffusion rate.

While Fick’s laws play a crucial role in describing diffusion in the context of pulmonary gas exchange, several factors influence the process. These factors include the partial pressure gradient of the gases, the surface area available for diffusion, the membrane’s thickness, and the gases’ diffusivity. These additional complexities make the final equations regulating the diffusion much more complicated. Mathematical and computational models are usually required to view the physiological process comprehensively.

## 5. Thoracic Endoscopy

Endoscopy in thoracic surgery utilizes advanced imaging techniques to visualize and access the thoracic cavity, allowing for minimally invasive procedures. Endoscopes are equipped with lenses that focus light and magnify images of internal structures. Modern endoscopy employs various imaging technologies to enhance visualization.

The minimally invasive incisions reduce pain, shorten recovery times, and lower the risk of complications. High-quality imaging allows for the better assessment and treatment of thoracic conditions. Lastly, the ability to manipulate instruments with high accuracy improves surgical outcomes.

Recent technological advancements in endoscopy have significantly improved outcomes in thoracic surgery. Integrating robotic systems into thoracic surgery has enhanced precision and control during procedures. This approach allows for more complex surgeries with minimal invasiveness, reducing complication rates, as well as improved quality of life and faster patient recovery times.

The trend indicates that robotic-assisted techniques may soon surpass traditional video-assisted thoracoscopic surgery (VATS) in adoption and effectiveness. Advances in imaging, such as high-definition cameras and 3D visualization, provide surgeons with more precise and detailed views of the thoracic cavity. This improved visualization aids decision making during surgery and enhances the overall safety and efficacy.

Although still in the early stages of adoption, Natural Orifice Transluminal Endoscopic Surgery (NOTES) represents a novel approach that allows surgeons to access the thoracic cavity through natural orifices, further minimizing invasiveness. This technique has shown promise in animal models and early human trials, potentially offering new avenues for thoracic interventions [15,16].

### The Physics Behind 

A fundamental law governing the behavior of light as it travels through different mediums is Snell’s law, which is also known as the law of refraction. When light travels from one medium to another, its speed changes due to the different optical densities of the two media. In a vacuum, light travels at the speed of *c*, the universal speed limit. But when light rays go through a medium, their speed decreases at *v*. The refractive index of the medium is therefore expressed as follows:

(7) $$n=\frac{c}{v}.$$

**Figure 1:**
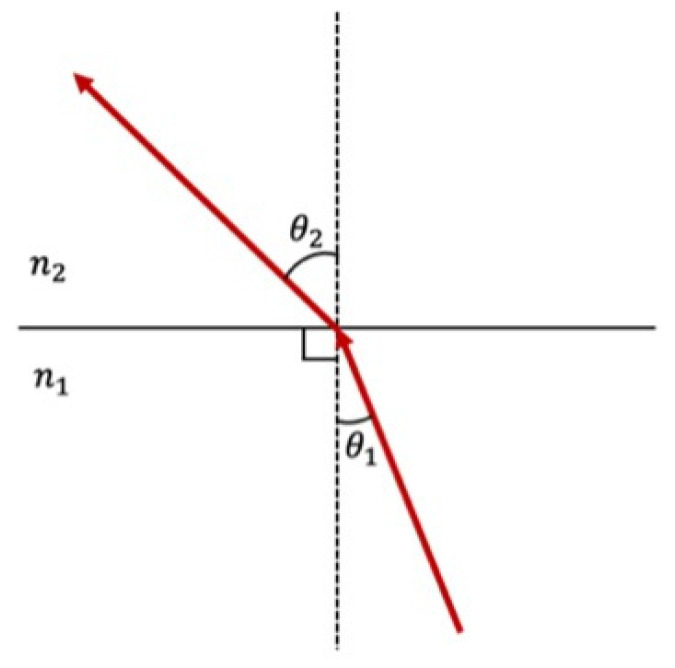
Snell’s law ($n_{1}sin(\theta_{1})=n_{2}sin(\theta_{2})$), where *θ*_1_ and *θ*_2_ are, respectively, the angle of incidence and the angle of refraction, and *n*_1_ and *n*_2_ are the refractive index of the two mediums.

While the change in light speed is not visible to the naked eye, the alteration in the light’s path can be observed as a change in direction. Snell’s law is mathematically expressed as follows:

(8) $$n_{1}sin(\theta_{1})=n_{2}sin(\theta_{2}),$$

where *θ*_1_ and *θ*_2_ are, respectively, the angle of incidence and the angle of refraction, and *n*_1_ and *n*_2_ are the refractive index of the two mediums (Figure 1). The design of endoscopic lenses relies on Snell’s law to control how light bends, as efficient light refraction helps to minimize distortions and enhances image clarity.

**Figure 2:**
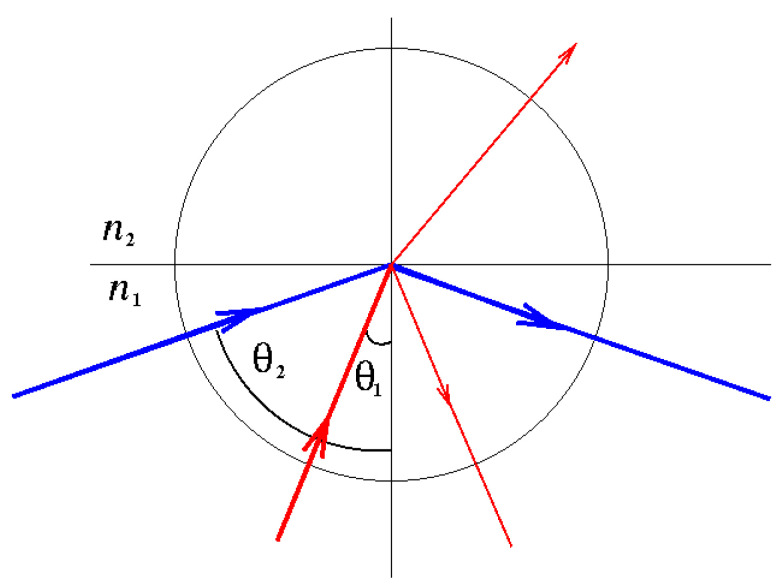
Total internal reflection. The incidence angle of the blue ray, *θ*_2_, is greater than the critical angle, so the light ray is completely reflected.

Optical fibers, essential components of endoscopes, must transmit light from its source into the body and return the reflected light back to the observer with minimal loss. To achieve this, optical fibers operate based on the principle of total internal reflection. Total internal reflection (Figure 2) occurs when light traveling within a medium of a higher refractive index (such as glass or fiber optic cables) strikes the boundary with a medium of a lower refractive index (such as air) at an angle greater than the critical angle. This causes the light to be completely reflected within the original medium without escaping, ensuring efficient light transmission with minimal loss.

## 6. Laser Technologies

Laser technology has become an increasingly valuable tool in thoracic surgery. It offers several advantages over traditional surgical methods, and its applications range from tumor resection to treating various thoracic conditions. Lasers provide high precision in cutting and coagulating tissue, minimizing damage to surrounding structures. This is particularly beneficial in delicate areas of the thoracic cavity. The coagulation effect of lasers helps to reduce bleeding during surgery. This is crucial in thoracic procedures where blood vessels are often involved. Patients typically experience less postoperative pain and a quicker recovery time compared to conventional surgical techniques. This is due to the minimally invasive nature of laser surgery, which often results in smaller incisions. Lasers can be used with VATS, enhancing the visualization of the surgical field and allowing for more effective interventions [17,18].

### The Physics Behind 

**Figure 3:**
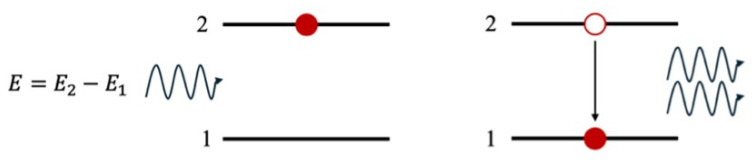
Depiction of the principle behind the stimulated emission of laser light. A photon with energy (*E*) = *E*_2_ − *E*_1_ can stimulate an electron to fall from the excited state (*E*_2_) (2) to the fundamental state (*E*_1_) (1). This process releases two photons of the same energy and in phase with each other.

LASER is an acronym for Light Amplification By Stimulated Emission of Radiation, which accurately describes its mechanism of action. Laser light operates through the direct application of quantum mechanics. Consider an active medium with two distinct energy levels, *E*_1_ and *E*_2_, where *E*_1_ < *E*_2_. If a greater number of electrons occupy the higher energy level (*E*_2_), they can be stimulated to transition to the lower energy level (*E*_1_) by a photon with energy (*E*) = *E*_2_ − *E*_1_. This process releases two photons of the same energy and in phase with each other, as shown in Figure 3.

In an active medium with a high concentration of electrons in the excited state (*E*_2_), the photons emitted by the transition of one electron can stimulate additional electron transitions, leading to a cascading process known as avalanche breakdown. The result is the emission of a large number of photons with identical characteristics, making the laser light highly directional, monochromatic (all photons have the same wavelength), and coherent (photons are in phase in time and space). These properties distinguish laser light from ordinary light beams, making it extremely effective for cutting and coagulating tissue.

An essential step in the generation of laser light involves creating a medium where more electrons are in the excited state (*E*_2_) than in the ground state (*E*_1_), a condition not naturally found in nature. This is because a fundamental principle of physics states that systems naturally tend towards the lowest-energy configuration. Therefore, to achieve an inverted population with more electrons in *E*_2_ than *E*_1_, a pumping system is required. The pumping system is a critical component of laser devices and determines the laser’s operational mode (pulsed or continuous).

Lasers can be classified based on the type of active medium: semiconductor lasers, gas lasers, solid-state lasers, and liquid lasers. In semiconductor lasers, stimulated emission is driven by the recombination of electron–hole pairs, with the pumping system delivering an electric current pulse to the semiconductor junction. In gas lasers, a gaseous mixture (e.g., CO_2_, He-Ne, Ar-Kr) serves as the active medium, and the pumping method involves an electrical discharge. In solid-state lasers, active atoms are introduced as impurities into a crystalline or glass matrix, and the pumping method is optical. Liquid lasers employ dye molecules dissolved in solvents (e.g., water or alcohol) as the active medium, with optical pumping as the excitation method.

**Figure 4:**
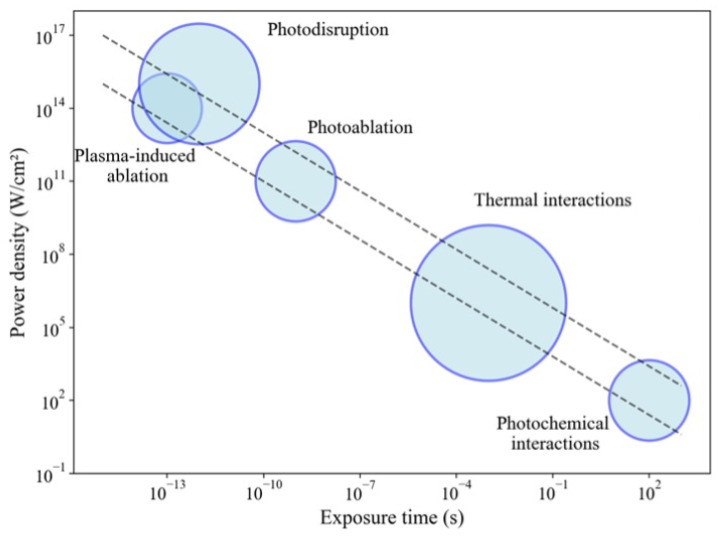
Different types of laser–tissue interactions based on exposure time (s) and power density (W/cm^2^).

Laser light interacts with tissue through various light–matter interactions. These effects are photochemical, photothermal, photodisruption, and photoablation effects. They occur depending on the duration of the exposure and the modality of the laser emission, as shown in Figure 4.

Photochemical interactions happen due to the presence of photosensitive molecules in human tissue. These interactions result in structural changes within the molecules, leading to the formation of the molecular structures. For these interactions to occur, the photons’ energy must exceed the chemical bonds’ binding energy between atoms and molecules.

Lasers operating via the thermal effect rely on light conversion into heat. At temperatures between 40 and 50 °C, we enter the hyperthermia range, where a few molecular bonds are disrupted, and cellular membranes are altered. This effect is reversible. At approximately 60 °C, protein and collagen denaturation occurs, leading to tissue coagulation and cellular necrosis. As the temperature reaches 100 °C, water within the tissue vaporizes, resulting in tissue cutting. When all the water molecules have evaporated, carbon residues are left behind, leading to the darkening of surrounding tissues and smoke release. This phenomenon is called carbonization. At temperatures around 300 °C and above, tissue melting and plasma formation can occur. In thoracic surgery, lasers are typically employed for their thermal effects.

The ablation effect is triggered when the tissue temperature reaches the boiling point of water. Small vapor bubbles form and become trapped within the tissue. As these bubbles expand and move, they exert a mechanical force. This process culminates in an explosive event, often called the “popcorn” effect.

Photomechanical disruption occurs at intensities so high that plasma is generated. The motion of this plasma creates spherical shock waves, which lead to localized mechanical ruptures at points where the increase in pressure surpasses the cohesive forces holding the tissue together.

This overview of light–matter interactions demonstrates that, if not handled correctly, laser light can cause significant damage not only to patient tissues but also to nearby operators.

## 7. Fluid Dynamics

Fluid dynamics plays a crucial role in various aspects of thoracic surgery. The pleural space between the visceral and parietal pleura is filled with a thin layer of pleural fluid. This fluid acts as a lubricant, allowing the lungs to slide smoothly against the chest wall during respiration. An abnormal accumulation of pleural fluid, known as pleural effusion, can occur due to various medical conditions. Understanding pleural fluid dynamics, including its production, absorption, and movement, is crucial for diagnosing and managing pleural effusions.

To begin with, understanding fluid dynamics principles is essential for positioning during thoracentesis, as the accumulation of pleural fluid creates a gradient that affects fluid distribution within the pleural cavity. This knowledge assists in selecting patient positioning that facilitates safe and efficient fluid removal. For instance, placing the patient in an upright, seated position is advantageous, as it allows the fluid to collect in the lower posterior pleural cavity, which is more easily accessible for drainage. Moreover, optimal ultrasound control points are crucial for guiding thoracentesis. Ultrasound enables the precise localization of pleural effusions, minimizes the risk of injuring surrounding structures, and helps identify the safest entry point. Using Doppler ultrasound, clinicians can monitor the fluid flow and predict changes in the pleural pressure, ensuring a controlled and gradual drainage that reduces the risk of complications, such as re-expansion pulmonary edema. Fluid dynamics principles play a role in planning drainage procedures, particularly when considering the speed and volume of the fluid removal. Rapid removal can disrupt the pleural pressure dynamics, leading to adverse outcomes. Therefore, knowledge of the fluid flow rate helps surgeons to plan controlled drainage intervals and maintain a steady pressure differential, optimizing patient outcomes.

The airflow dynamics within the respiratory system is essential for maintaining proper lung function. Fluid dynamics helps understand the behavior of air as it moves through the airways during inhalation and exhalation. This knowledge is critical in managing airway obstruction, where airflow is impeded, and in designing ventilation strategies for patients undergoing thoracic surgery.

The cardiovascular system is closely related to the thoracic cavity, and fluid dynamics plays a significant role in understanding its function. The blood flow behavior within the heart and major blood vessels, such as the aorta and pulmonary arteries, is crucial for assessing cardiovascular health and planning surgical interventions. Fluid dynamics helps diagnose and manage conditions like aortic aneurysms, dissections, and valvular heart diseases.

Fluid dynamics also influences the design and use of surgical instruments and techniques in thoracic surgery. For example, understanding the behavior of fluids during minimally invasive procedures, such as VATS, helps develop specialized instruments and methods that minimize tissue damage and optimize surgical outcomes.

Fluid dynamics plays a critical role in preventing postoperative complications in thoracic surgery. Understanding fluid dynamics helps surgeons effectively manage pleural effusions and other fluid accumulations in the thoracic cavity. By applying principles of fluid movement, surgeons can optimize drainage techniques, such as thoracentesis or chest tube placement, to ensure the efficient removal of excess fluid. Fluid dynamics is essential in designing ventilation strategies that minimize lung injury during and after surgery. Protective lung ventilation techniques, considering the airflow dynamics and pressure within the lungs, can help prevent ventilation-induced and acute lung injury. A solid understanding of fluid dynamics allows for the better management of hemodynamics during surgery. Surgeons can monitor blood flow and pressure changes in real time, anticipating potential complications such as bleeding or cardiac instability. Fluid dynamics principles facilitate the effective monitoring of the fluid movement in the thoracic cavity post-surgery. Knowledge of fluid dynamics informs the choice of surgical techniques and instruments, particularly in minimally invasive procedures [19,20,21,22].

### The Physics Behind 

Poiseuille’s law provides a mathematical relationship for calculating the volumetric flow rate of a fluid given certain conditions. Poiseuille’s law states that the volumetric flow rate (*Q*) of an incompressible, Newtonian fluid through a long, straight, cylindrical pipe is directly proportional to the fourth power of the pipe’s radius (*r*) and the pressure difference (Δ*P*) along the length of the pipe and is inversely proportional to the fluid’s viscosity (*η*) (which is the fluid internal friction) and the length (*L*) of the pipe. Its mathematical expression is the following:

(9) $$Q=\frac{\pi r^{4}\Delta P}{8\eta L}.$$

*Poiseuille’s law* is only applicable in specific conditions, such as Newtonian fluids and laminar flows, which is exactly the case of blood, so Poiseuille’s law helps to understand how blood flows can be used to perform simple calculations to evaluate the blood flow rate in specific vessels. For instance, increasing the radius of a vessel from 1 mm to 2 mm can result in a 16-fold increase in the blood flow.

The *continuity equation* is another fundamental principle in fluid dynamics with practical applications in surgery. Based on the conservation of mass, this equation states that if the cross-sectional area of a vessel decreases, the flow velocity increases. It is expressed as follows:

(10) $$A_{1}V_{1}=A_{2}V_{2},$$

where $A_{1}$ and $A_{2}\;$ are the cross-sectional areas at two points in the vessel, and $V_{1}$ and $V_{2}\;$ are the corresponding flow velocities. While this equation ideally applies to incompressible, non-viscous fluids, it provides a reasonable approximation for blood flow in vessels.

*Bernoulli’s principle* is a renowned fluid dynamics principle derived from the conservation of energy in a fluid flow system. Bernoulli’s principle states that in a streamlined flow of an incompressible, non-viscous fluid, the total mechanical energy of the fluid remains constant. This energy is the sum of the fluid’s kinetic energy, potential energy, and pressure energy. Its mathematical form is the following:

(11) $$P+\frac{1}{2}\rho v^{2}+\rho gh=cost,$$

where *P* is the fluid pressure, *ρ* is the density, *v* is the velocity, *g* is the acceleration due to gravity, and *h* is the height above the reference level. This equation reflects the conservation of mechanical energy within a fluid system. A key implication of Bernoulli’s principle is that an increase in the fluid velocity leads to a decrease in pressure.

The *Doppler effect* describes the change in the frequency or wavelength of a signal in relation to an observer moving relative to the source of the signal. Commonly experienced with sound waves—such as the change in pitch of an ambulance siren as it passes by—the Doppler effect is also applied in Doppler ultrasound technology. The Doppler effect and Bernoulli’s principle are implemented in Doppler ultrasound: by analyzing the shift in the frequency of the reflected ultrasound waves, the imaging system calculates the velocity and pressure changes in the blood flow.

*Laplace’s law* relates to fluid mechanics and describes the relationship between the pressure difference across a curved surface and the curvature of that surface. Laplace’s law relates the pressure difference across the wall of a spherical or cylindrical structure to the tension or stress in the wall and the curvature of the structure. For a cylindrical structure, as a blood vessel can be, it can be written as follows:

(12) $$\Delta P=\frac{T}{r},$$

where ΔP is the pressure difference across the wall, *T* is the surface tension or wall tension, and *r* is the radius of the sphere.

*Dalton’s law* states that the total pressure ($P_{total}$) exerted by a mixture of gases is equal to the sum of the partial pressures of its individual gas components ($P_{1},P_{2},\ldots ,\;P_{n})$. Mathematically, it can be expressed as follows:

(13) $$P_{total}=P_{1}+P_{1}+P_{3}+\ldots +P_{n}.$$

*Henry’s law* states that the amount of a gas that dissolves in a liquid is directly proportional to the partial pressure of that gas above the liquid:

(14) $$C=K_{H}\times P,$$

where *C* is the concentration of the gas dissolved in the liquid, $K_{H}$ is Henry’s law constant for the specific gas–liquid pair, and *P* is the partial pressure of the gas.

These two principles have several implications in thoracic surgery, especially in oxygenation and gas exchange. For instance, the solubility of gases in blood and tissues, as described by Henry’s law, affects the efficiency of the gas exchange in the lungs. This is crucial for assessing how well gases like oxygen and carbon dioxide are being transported and exchanged in patients with respiratory issues.

## 8. Discussion

The intersection of physics and thoracic surgery represents a crucial domain where advanced scientific principles directly inform and enhance surgical practice. Thoracic surgery involves intricate procedures within the chest cavity and requires a profound understanding of the physical laws governing the tools, techniques, and biological processes. Electrosurgery and electrocoagulation are other critical areas where physics is integral to thoracic surgery. These techniques rely on the principles of electrical currents and thermal energy governed by Ohm’s law (Equation (3)) and Joule’s effect (Equation (4)). The ability to precisely control the current flow through tissues, thereby generating heat to cut or coagulate, is essential for minimizing collateral damage in the thoracic cavity, where delicate structures such as blood vessels and nerves are at risk. The distinction between monopolar and bipolar electrosurgery reflects the ongoing evolution of these techniques, with the latter offering enhanced precision and safety. Surgical interventions, such as lung resections, can significantly alter the diffusion dynamics, necessitating a careful consideration of the remaining lung tissue’s capacity to maintain adequate gas exchange.

Moreover, the management of the ventilation–perfusion balance is crucial for optimizing patient recovery. Thoracic endoscopy, which has revolutionized minimally invasive surgery, relies heavily on optics and light behavior principles, particularly Snell’s law of refraction (Equations (7) and (8)). Visualizing internal structures with high precision is fundamental to the success of endoscopic procedures. Advances in endoscopic technology, such as high-definition imaging and 3D visualization, have significantly improved the accuracy and safety of thoracic surgery. Integrating robotic systems further enhances the surgeon’s ability to perform complex procedures with minimal invasiveness, reducing patient morbidity and accelerating recovery. Fluid dynamics is another critical area where physics informs thoracic surgical practice. The principles of fluid flow, as described by Poiseuille’s law (Equation (9)), Bernoulli’s principle (Equation (11)), and the continuity equation (Equation (10)), are essential for understanding blood flow, respiratory mechanics, and the movement of pleural fluids. These principles guide the management of conditions such as pleural effusions, airway obstructions, and cardiovascular complications during thoracic surgery.

Understanding fluid dynamics is essential for procedures involving pleural effusions, where strategic management can alleviate symptoms and prevent complications. For example, fluid dynamics principles guide thoracentesis planning, from determining the patient positioning to selecting the optimal points for ultrasound guidance. Effective patient positioning can enhance gravitational fluid drainage, thus allowing for more efficient and safer fluid removal. Additionally, identifying ultrasound landmarks informed by fluid dynamics improves accuracy in needle placement, minimizing risks such as pneumothorax and maximizing the therapeutic benefit.

In thermodynamics, energy-based surgical tools—such as ultrasonic scalpels and electrocautery devices—rely on precise heat transfer and energy distribution to function effectively without damaging surrounding tissues. Understanding thermodynamics helps surgeons tailor tool settings to prevent excess thermal injury, especially in thoracic procedures where tissue sensitivity is paramount. These principles allow for fine tuning energy levels, facilitating controlled coagulation while preserving the structural integrity of the surrounding pulmonary and vascular tissues.

The application of mechanics is also evident in tissue stapling and suturing, where force distribution plays a pivotal role in reducing tissue trauma. The mechanics of staple application, for instance, inform the device selection and angle of application, ensuring even force across tissues, which is particularly important when dealing with fragile or compromised lung tissue. By understanding these forces, surgeons can select stapling devices and techniques that minimize shear stress and promote smoother healing and fewer postoperative complications.

### Limitations

Several limitations should be acknowledged. Firstly, the manuscript focuses on established physical principles and their direct applications to thoracic surgical techniques and technologies. Secondly, the manuscript’s emphasis on the practical applications of physics in thoracic surgery may not delve deeply into the underlying theoretical physics that could further inform and innovate surgical practices. Another limitation is the relatively broad scope of the manuscript, which may result in a need for more detailed discussions of specific surgical procedures or technologies. By covering a wide range of physical principles and their applications, this manuscript might sacrifice depth for breadth, potentially leaving out detailed technical insights that could be valuable to thoracic surgeons. Lastly, this manuscript may not fully address the interdisciplinary collaboration required to apply physics in thoracic surgery effectively. While the importance of understanding physical principles is emphasized, the role of cooperation between surgeons, physicists, engineers, and other specialists in advancing thoracic surgery is not extensively discussed. This collaboration is crucial for developing and refining technologies that integrate physics into surgical practice.

## 9. Conclusions

Physics has profoundly influenced the evolution and practice of thoracic surgery. By elucidating critical physical principles and their application to various surgical techniques and technologies, it is evident that a deep understanding of physics is indispensable for advancing thoracic surgical practices. From the precise application of fluid dynamics in cardiothoracic procedures to the critical role of radiation physics in developing imaging technologies, the integration of physics has enhanced thoracic surgeries’ safety, precision, and outcomes.

As the field continues to evolve, embracing interdisciplinary collaboration and staying attuned to emerging physical theories will be essential in pushing the boundaries of what is possible in thoracic surgery. Future innovations will likely stem from this intersection of disciplines, promising even more sophisticated and effective surgical interventions. Therefore, the continuous exploration of the physical sciences within the surgical realm is beneficial and necessary for improving patient care in thoracic surgery.
